# Applications of Raman spectroscopy in the diagnosis and monitoring of neurodegenerative diseases

**DOI:** 10.3389/fnins.2024.1301107

**Published:** 2024-02-02

**Authors:** Chao Chen, Jinfeng Qi, Ying Li, Ding Li, Lihong Wu, Ruihua Li, Qingfa Chen, Ning Sun

**Affiliations:** ^1^Central Laboratory, Liaocheng People’s Hospital and Liaocheng School of Clinical Medicine, Shandong First Medical University, Liaocheng, China; ^2^Department of Geriatrics, Tianjin Medical University General Hospital, Tianjin Geriatrics Institute, Tianjin, China; ^3^Department of Clinical Laboratory, Liaocheng People’s Hospital and Liaocheng School of Clinical Medicine, Shandong First Medical University, Liaocheng, China; ^4^Institute of Tissue Engineering and Regenerative Medicine, Liaocheng People’s Hospital and Liaocheng School of Clinical Medicine, Shandong First Medical University, Liaocheng, China; ^5^Research Center of Basic Medicine, Jinan Central Hospital, Jinan, China

**Keywords:** Raman spectroscopy, neurodegenerative diseases, Alzheimer’s disease, Parkinson’s disease, Huntington’s disease, amyotrophic lateral sclerosis

## Abstract

Raman scattering is an inelastic light scattering that occurs in a manner reflective of the molecular vibrations of molecular structures and chemical conditions in a given sample of interest. Energy changes in the scattered light can be assessed to determine the vibration mode and associated molecular and chemical conditions within the sample, providing a molecular fingerprint suitable for sample identification and characterization. Raman spectroscopy represents a particularly promising approach to the molecular analysis of many diseases owing to clinical advantages including its instantaneous nature and associated high degree of stability, as well as its ability to yield signal outputs corresponding to a single molecule type without any interference from other molecules as a result of its narrow peak width. This technology is thus ideally suited to the simultaneous assessment of multiple analytes. Neurodegenerative diseases represent an increasingly significant threat to global public health owing to progressive population aging, imposing a severe physical and social burden on affected patients who tend to develop cognitive and/or motor deficits beginning between the ages of 50 and 70. Owing to a relatively limited understanding of the etiological basis for these diseases, treatments are lacking for the most common neurodegenerative diseases, which include Alzheimer’s disease, Parkinson’s disease, Huntington’s disease, and amyotrophic lateral sclerosis. The present review was formulated with the goal of briefly explaining the principle of Raman spectroscopy and discussing its potential applications in the diagnosis and evaluation of neurodegenerative diseases, with a particular emphasis on the research prospects of this novel technological platform.

## Introduction

Raman spectroscopy is a developed analytical approach that can enable the noninvasive characterization of biological samples with a high degree of spatial resolution. In this process, an incident laser is used to excite samples, at which time both Rayleigh (elastic) and Raman (inelastic) scattering can occur. The difference in energy between the incident photon and the Raman scattering photon corresponds to the chemical bond excited by this energy, and it can be readily quantified by assessing differences between the excitation wavelength and the emitted wavelength of light. Raman spectroscopy can thus permit the effective characterization of various vibrational modes associated with the chemical bonds that comprise a given sample (Gaba et al., 2022). Recent studies have emphasized the utility of Raman spectroscopy in the context of biomedical research, providing a unique opportunity to diagnose a range of diseases (Ralbovsky and Lednev, 2020). Relative to other imaging modalities, noninvasive Raman spectroscopy offers advantages including low levels of signal background, a high degree of spatial resolution, a high degree of chemical specificity, excellent optical stability, and the ability to detect multiple targets (Ramya et al., 2021).

Neurodegenerative diseases are debilitating conditions that result in the progressive loss of neuronal function in the peripheral and/or central nervous system, ultimately causing long-term cognitive and/or motor impairment in affected patients (Fang et al., 2020). The most commonly diagnosed neurodegenerative diseases include Alzheimer’s disease (AD), Parkinson’s disease (PD), Huntington’s disease (HD), and amyotrophic lateral sclerosis (ALS). Patients suffering from these diseases experience a range of debilitating physical and mental symptoms that are determined based on the types of neurons undergoing degeneration and the spatial organization thereof. AD, for example, is characterized by cognitive impairment that results from pyramidal neuron degeneration within the cerebral cortex, whereas PD patients experience motor symptoms that stem from the loss of dopaminergic neurons in the substantia nigra (Bloomingdale et al., 2022). Labeled Raman imaging strategies have been developed to gain highly sensitive and selective subcellular insights. When effectively optimized and implemented, this combination of targeted labeling and Raman analysis can specifically focus on organelles of interest, enhancing the obtained Raman spectrum to send signals or produce strong peak bands (1800–2,800 cm^−1^) within the biologically Raman silent spectral region (Adamczyk et al., 2021). Raman cell imaging can offer insight into the chemical composition of biological samples, providing new opportunities for the detection and diagnosis of neurodegenerative diseases (Huang et al., 2021, 2023; Sharma et al., 2022).

Raman spectroscopy (RS) has great potential for the detection of new biomarkers, which can assist the clinical screening of disease susceptibility and determination of the incidence rate. Compared with conventional chemical analytical techniques, RS has the advantages of being non-destructive, fast, environmentally friendly, with no need for sample preparation, capable of working on small samples with low consumption of chemical reagents, and high repeatability, making it suitable for the rapid detection of target substances. Additional advantages are that it does not cause bleaching or flickering, and does not show self-quenching (Liu et al., 2020). RS can also preserve medical samples under almost physiological conditions and can be used for live samples, providing the possibility of diagnosis in real-time diagnosis, which other techniques do not permit. RS includes developed surface enhanced Raman spectroscopy (SERS), tip enhanced Raman spectroscopy (TERS), Fourier-transform Raman spectroscopy (FT Raman), resonance Raman scattering (RRS), and dry anti-Stokes Raman scattering (CARS). These allow the non-invasive evaluation of biological specimens such as blood, urine, cerebrospinal fluid, and saliva, and can be used for minimally invasive and real-time monitoring of tissue *in vivo*, assisting in the clinical diagnosis of neurodegenerative diseases, such as Alzheimer’s disease, Parkinson’s disease, and amyotrophic lateral sclerosis (Mammadova et al., 2019).

## The current status of neurodegenerative disease diagnostics

The progressive aging of the global population is driving an increase in the prevalence of debilitating neurodegenerative diseases that adversely impact patient quality of life while imposing a significant economic and societal burden (Meng et al., 2021). Aging is the primary risk factor associated with AD, PD, and other neurodegenerative diseases, with an estimated 10% of adults ≥65 years old exhibiting AD and further increases in these rates with advanced age. At present, effective treatment options for aging-related neurodegenerative diseases are limited, and patients typically experience irreversible disease progression (Hou et al., 2019). Neuronal integrity is highly dependent on mitochondrial ATP synthesis, metabolite synthesis, and synaptic calcium binding. Axons, owing to their extreme length, require reliable retrograde and anterograde mitochondrial transport to and from synapses in order to ensure that nerve transmission can occur effectively through membrane potential generation. Mitochondrial dysfunction is thus a hallmark of many neurodegenerative diseases. These conditions are also commonly characterized by pathogenic protein aggregation, although the mechanistic basis for this observation remains uncertain (Palmer et al., 2019). Under physiological conditions, certain mislocalized or aggregated pathological proteins associated with neurodegenerative disease have been shown to undergo liquid–liquid phase separation (LLPS). These phase transitions may serve an important physiological role, with toxicity potentially resulting from disease-related changes in this LLPS behavior as a consequence of mutations or other adverse changes. Treatment strategies that seek to interfere with aberrant LLPS may thus be capable of abrogating the aggregation and toxicity that are closely associated with neurodegeneration (Darling and Shorter, 2021). While protein misfolding is believed to be an important underlying factor in many forms of neurodegenerative disease, the precise targets responsible for the regulation of these proteins and the modulation of downstream effects are incompletely understood.

**Figure 1:**
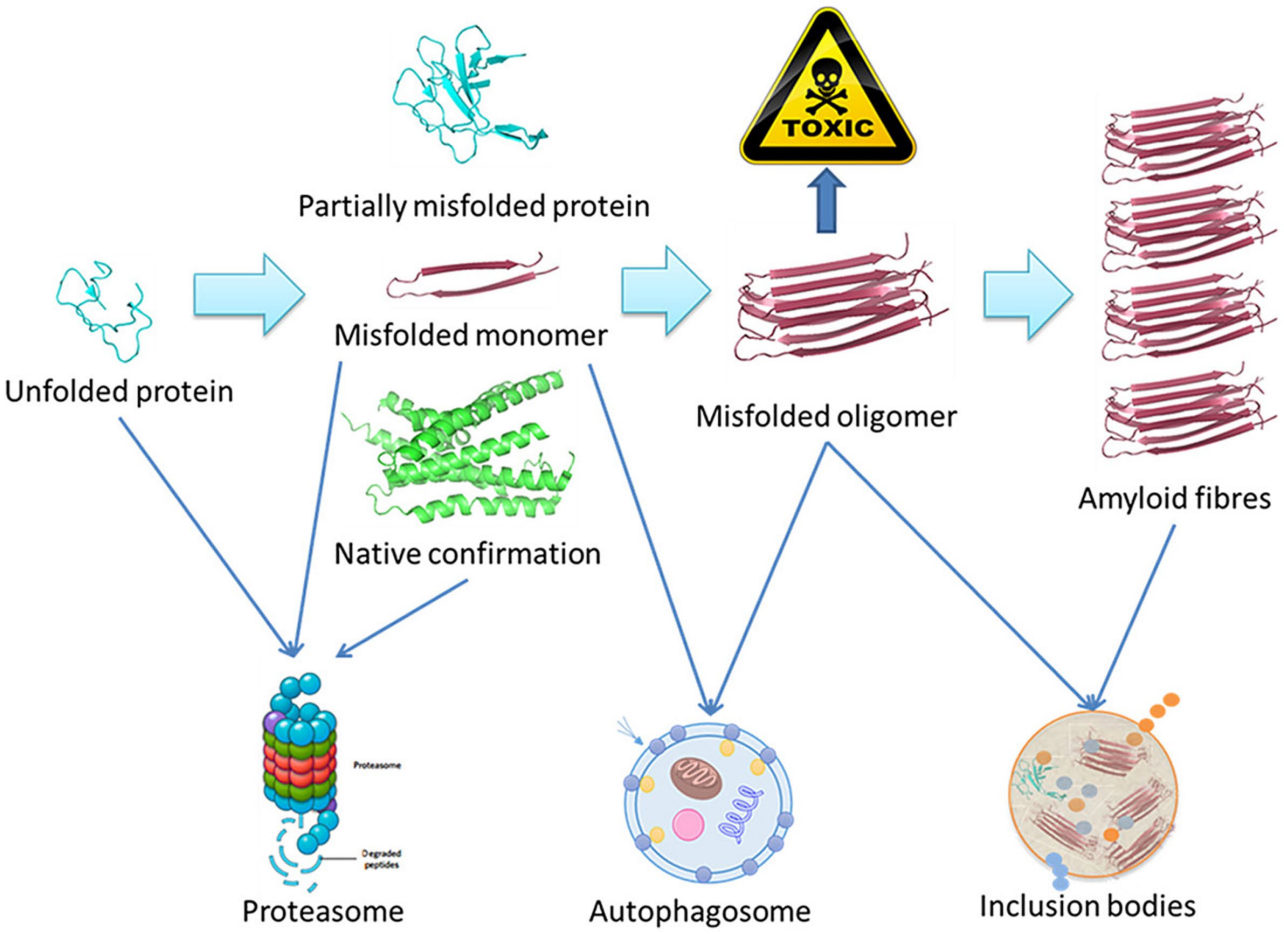
Schematic overview of the misfolded protein degradation process (Ma et al., 2019).

Using amyloid fibrils as an example, Figure 1 presents a schematic overview of the degradation of misfolded proteins. A range of molecular size-dependent mechanisms can facilitate the elimination of these misfolded and aggregated proteins. When mutations, disease-related conditions, or other factors lead to the misfolding of these proteins, if they are not degraded, they instead undergo processing into small misfolded oligomers that include α-synuclein, β-amyloid, and multi-Q protein. Cells employ proteolytic activity, autophagy, and the formation of inclusion bodies as protective mechanisms aimed at eliminating or sequestering proteins that are misfolded or aggregated. Proteasomal degradation can generally eliminate any misfolded or unfolded proteins when they are in a monomeric state, while most misfolded oligomers can be eliminated via autophagy.

Phenotypic screening provides an unbiased approach to screening for novel targets and therapeutic compounds using a range of systems including model animals and human cell lines. While these models can recapitulate many aspects of disease-related biology, they are inherently limited. Through the further improvement of the sensitivity and accuracy of these screening techniques, it may be possible to more readily detect misfolded proteins such that neurodegenerative diseases can be detected in the earliest stages of development (Brown and Wobst, 2020).

**Figure 2:**
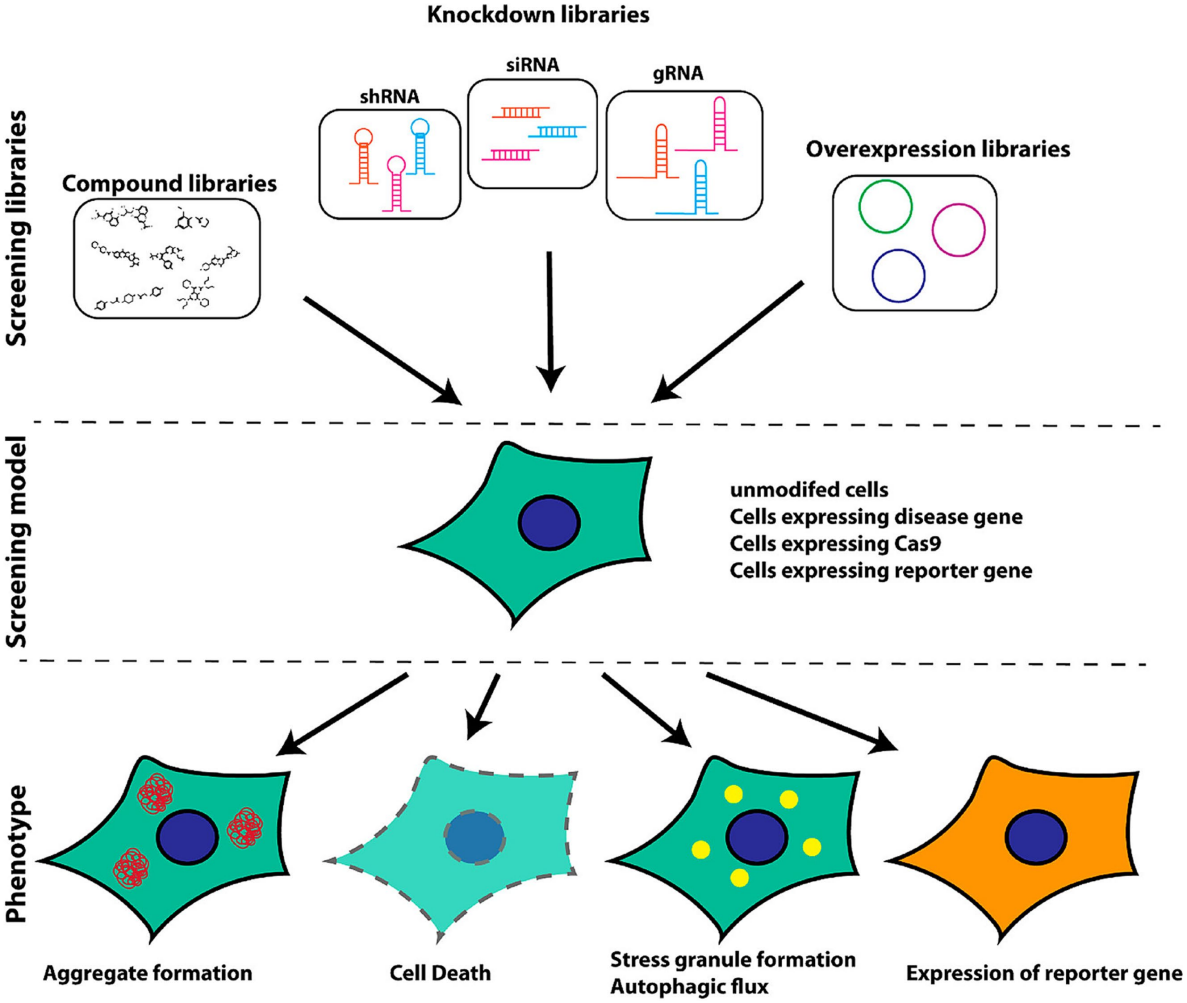
Schematic overview of the selection of appropriate models for cellular screening based on library type and phenotypes of interest (Brown and Wobst, 2020).

**Figure 3:**
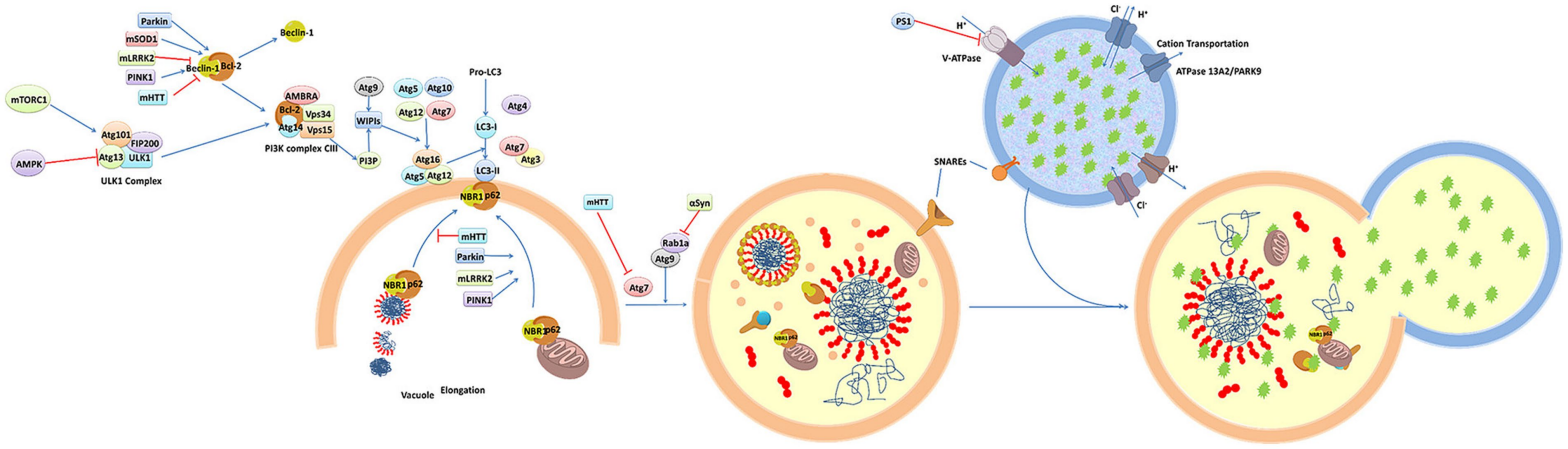
An overview of the molecular dynamics of autophagy (Ma et al., 2019).

Figure 2 presents a schematic overview of the phenotypic screening method. Initially, an appropriate cellular model is selected based on the phenotype of interest and the screening library being employed These libraries can include small molecule libraries, knockdown/deletion libraries, and overexpression libraries. Target cells generally express genes that contribute to the incidence of a given disease-related phenotype, such as the overexpression of synaptic nucleoprotein, tau, or other relevant targets. Cells rely on a series of quality control mechanisms that monitor the status of proteins and organelles so as to maintain homeostatic balance. These mechanisms include the molecular chaperone-mediated folding of proteins and the ability to induce proteasomal degradation or autophagic activity as appropriate. The autophagic process is outlined in Figure 3. Mutations or other factors that impair autophagy are commonly observed in the context of neurodegenerative disease, with abnormal interactions between Beclin 1 and mutant Huntington (mHTT) protein, superoxide dismutase 1 (mSOD1), LRRK2, Parkin, and PINK1 potentially disrupting the initiation of this process. Both PINK1 and parkin are vital to the effective elimination of damaged mitochondria, and this process of mitophagy is frequently dysfunctional in PD and other diseases owing to mutations or other changes that impact these proteins. mHTT expression can also contribute to altered vesicle recognition that compromises normal autophagic activity, while α-synuclein can interact with Rab1a to suppress this process. Presenilin-1 (PS1) mutations can lead to abnormal lysosomal acidification and autophagy-associated damage, and there is growing evidence that the disruption of autophagic activity is associated with the formation of inclusion bodies containing misfolded proteins that are hallmarks of many neurodegenerative diseases. Mutations of core autophagy-associated genes have been closely linked to AD, PD, HD, and other forms of disease (Park et al., 2020).

Stem cells and stem cell-derived factors have been widely studied as promising tools for the treatment of neurodegeneration, facilitating reduced neuroinflammation, myelin regeneration, and functional recovery following injury in some reports. The precise pathways whereby stem cells can exert these neuroprotective benefits, however, remain to be clarified and are thought to be related to the ability of these cells to secrete an array of factors and extracellular vesicles (Fayazi et al., 2021). Gene therapy strategies have emerged as an attractive means of correcting etiological abnormalities that underlie a given disease state in a persistent manner, providing a promising alternative to the underwhelming performance of more traditional pharmacological interventions in the neurodegenerative disease space. Viral vector-based gene therapies have recently achieved great success in the management of spinal muscular atrophy, preserving motor function and improving survival following a single intravenous injection. While limitations to the development of gene therapy strategies persist, the advent of novel viral vectors capable of delivering genes to the central nervous system may provide an unprecedented opportunity to manipulate disease-related pathways through advanced genomic engineering (Schartz and Tenner, 2020; Sun and Roy, 2021).

Owing to their high energy demands, high levels of transcriptional activity, and prolonged lifespan, neurons are highly sensitive to DNA damage accumulation. While recent reports have suggested that DNA damage and mutations may contribute to neuronal diversity in the context of development and shape neuronal functions throughout life, there is also strong evidence that a range of neurological disease, including aging-related neurodegenerative diseases, are closely associated with DNA damage and defective DNA damage repair (Welch and Tsai, 2022). The characteristics of many neurodegenerative diseases are highly similar, and factors such as inflammation, excitotoxicity, oxidative stress, and neuron loss are thought to play a role in many of these conditions (Hrelia et al., 2020).

Recently, blood biomarkers have been proposed for the detection of neurodegenerative diseases, some of which are capable of detecting early-stage disease. The accurate identification and diagnosis of Alzheimer’s disease (AD), especially early AD, has important clinical significance and can assist with early intervention. To date, the differentiation between AD and Lewy body dementia (DLB) remains challenging and many cases of the latter are misdiagnosed as AD. A study by Paraskevaidi et al. investigated the diagnosis of early- and late-stage AD and DLB, as well as the differentiation between AD and DLB. The study recruited 56 individuals and divided them into four groups, namely, early AD, late AD, DLB, and healthy controls. RS was used to analyze the plasma samples from patients with early AD, late AD, and DLB, and healthy controls. The results showed that the classification algorithm achieved high accuracy for the different groups. For example, compared with healthy individuals, the early AD group showed a sensitivity of 84% and a specificity of 86%, while late-stage AD samples showed a sensitivity of 84% and a specificity of 77%. The sensitivity and specificity of distinguishing between early- and late-stage AD were 66 and 83%, respectively. The overall performance of the classification model was satisfactory. The results indicated that RS using blood samples was highly effective in distinguishing between samples from patients with AD and healthy individuals, as well as between two different types of dementia (Paraskevaidi et al., 2018). Extracellular vesicles (EVs) have been used as novel biomarkers for PD due to their role as carriers for various PD-related molecules; however, there are technical limitations in their detection and characterization in clinical settings. Gualerzi et al. proposed a Raman-based approach for the label-free analysis of cyclic EVs as a diagnostic and predictive tool for PD. This study recruited 40 subjects, including 18 healthy individuals and 22 patients with idiopathic PD. After purification of the vesicles from the sera of PD patients and healthy subjects, the EVs were analyzed using RS. Descriptive and multivariate statistical analyses of the RS data were performed, showing a 71% accuracy of RS in distinguishing between PD patients and healthy volunteers through the spectral fingerprint of cyclic EVs. The results showed that RS analysis of cyclic EVs couls clearly distinguish PD patients from healthy subjects of the same age (Gualerzi et al., 2019). Huntington’s disease (HD) is a neurodegenerative disease caused by multiple CAG (cytosine adenine guanine) repeats in the huntingtin (*HTT*) gene encoding the huntingtin protein Htt. This protein is associated with the dysregulation of cellular components in peripheral fibroblasts, which form part of the lipid raft in the plasma membrane subdomain. Therefore, HD may have an impact on the biochemistry and structure of cell membranes, and plasma membrane analysis may be a useful diagnostic biomarker. Muratore applied RS and partial least squares (PLS) chemometric methods to preliminarily confirm that the parenchymal membrane is indeed a suitable subcellular biomarker for the indirect detection of HD. This study analyzed the spectral regions between 400 and 1800 cm^−1^ and 2,700 and 3,200 cm^−1^, with the previous region showing the most significant differences and peak shifts between the plasma membranes extracted from HD and control fibroblasts. The main difference in the plasma membrane composition was found to lie in subcellular components associated with cholesterol, phospholipids (mainly phosphatidylinositol), and proteins containing tyrosine. These findings indicate that extracted plasma membranes, together with RS analysis, could be a useful biomarker for distinguishing HD in peripheral cells (Muratore, 2013). Picardi et al. compared the Raman spectral characteristics of spinal cord tissue sections from transgenic, ALS model mice, and non-transgenic mice using a 457-nm excitation line, and obtained strong Raman signals and detailed spectra in the molecular fingerprint region from the tissue sections. This study analyzed cross-sections of the spinal cords four SOD1G93A mice 75 days after birth, together with two SOD1G93A mice 90 days after birth, and compared them with sections from control mice of similar ages to evaluate the potential of RS at 457 nm excitation for the early detection of degenerative disease. The results showed that the spectra obtained from the gray matter of diseased mice significantly different from those from healthy mice. Notably, the intensity of the spectral windows 450–650 cm^−1^ and 1,050–1,200 cm^−1^ increased, while the intensities of the lipid contribution decreased at ~1,660 cm^−1^,~1,440 cm^−1^, and ~ 1,300 cm^−1^. At the biomolecular level, the observed spectral changes are associated with the degradation of lipid structures, axonal demyelination, and the accumulation of invasive proteoglycans, all of which are indicative of neurodegeneration. Based on these results, it is recommended to use the relative intensity of Raman signals in the spectral windows 450–650 cm^−1^ and 1,050–1,200 cm^−1^ as ALS markers in the spinal cord for early disease detection (Picardi et al., 2018).

## Raman spectroscopy: an overview

**Figure 4:**
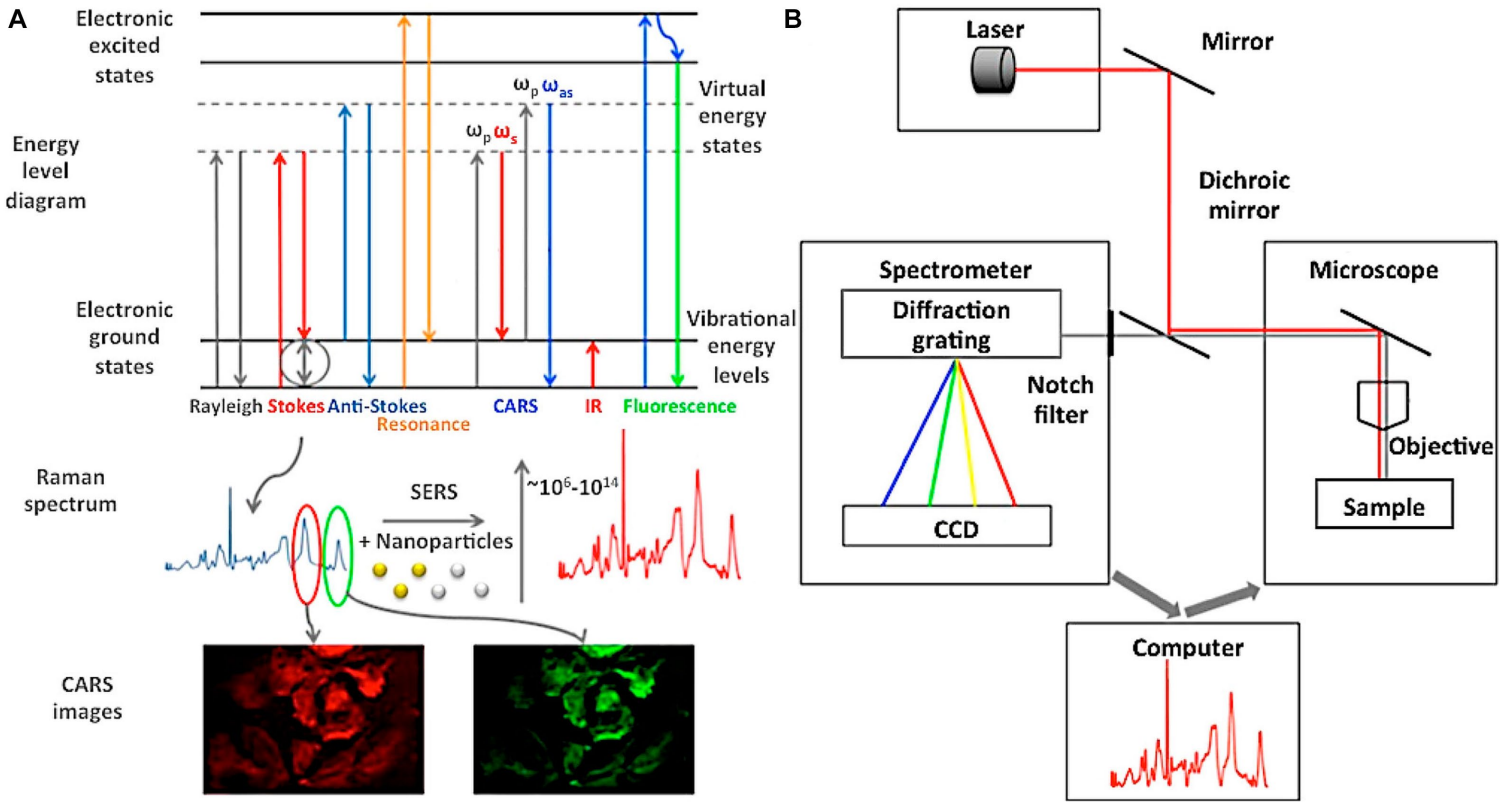
An overview of the general settings used for Raman microscopic spectroscopy systems (Devitt et al., 2018). **(A)** Energy level diagram of Raman spectroscopy. **(B)** Generic settings for Raman microscopic spectroscopy systems.

Raman spectroscopy is a technique that can be applied for material characterization, chemical sensing, and biological imaging, drawing extensive interest from a range of fields (Table 1). The Raman effect results from inelastic light scattering, providing a means of directly assessing the rotational vibration state of particular molecules and materials (Jones et al., 2019). An overview of the general arrangement of a Raman microspectral system is provided in Figure 4. In general, a laser is used to excite vibrations in target molecules, inducing spontaneous Raman scattering. The scattered light can then be collected at angles of 90° or 180°, and a filter at the wavelength of the incident light can be used to remove the Rayleigh scattering. A diffraction grating can then separate the light signal into separate frequencies that are then converted into electrons with a charge-coupled device (CCD) or other detector. Through the coupling of the spectrometer and a computer, digitized information can be collected in a spectral form. Light can also be focused on targets through the coupling of a Raman spectrometer and a microscope. Raman spectroscopy has been a focus of growing interest owing to the portable, specific, rapid, and non-destructive nature of this molecular fingerprinting technology (Qu et al., 2022). While some approaches to the imaging of biological macromolecules including proteins and nucleic acids have long been established, microscopy-based techniques are subject to inherent limitations, particularly when assessing smaller molecules that are nonetheless biologically relevant. Recent advances in Raman imaging, including the design of microscopes and customized vibration labels, have provided new opportunities to non-invasively evaluate small and intermediate-sized biomolecules within live cells, tissues, or organisms (Shen et al., 2019).

**Table 1:** Summary of different Raman methods as diagnostic tools for different neurodegenerative diseases.

Raman spectroscopy	Characteristics	Advantages	Disadvantages	Applications
Surface Enhanced Raman Spectroscopy	Using metal as a substrate to enhance signals	High sensitivity, multiple detections, low required sample concentration, non-destructive	Few substrate options and difficult to control stability	Early diagnosis and treatment of Huntington’s disease; Early detection of biomarkers for Alzheimer’s disease; Early detection of amyotrophic lateral sclerosis
FT-Raman spectroscopy	The coupling of a diode pumped neodymium doped yttrium aluminum garnet (Nd: YAG) laser with a Michelson interferometer produces	Non invasive, fast, and relatively inexpensive surgery with high overall signal-to-noise ratio	N/A	The possibility of *in vivo* diagnosis of Alzheimer’s disease
Confocal Raman microscopy spectroscopy	Artificial neural network discrimination of Raman signal differences	Non-Invasive	N/A	AD differential diagnosis Stratification of Parkinson’s disease patients, evaluation of rehabilitation and drug treatment outcomes.
Optical tweezers Raman spectroscopy technology	Combining laser tweezers to study individual cells in suspension	Fast, non-destructive, high specificity, and sensitivity	High requirements for lasers	Diagnosis of Alzheimer’s disease
Traditional Raman spectroscopy technology	The frequency of scattered photons is different from that of incident photons	The system is relatively simple, sensitive, unmarked, and non-destructive	low sensitivity	Alzheimer’s disease screening
Coherent anti Stokes Raman spectroscopy	Two incident light beams irradiate the sample and interact with each other, producing anti Stoke markings	Signal enhancement, no need for fluorescent labeling	The equipment is expensive and has a strong non resonant background	Early non-invasive diagnosis of Alzheimer’s disease

Proteins are large and highly complex, and while properly folded proteins are essential to all physiological processes, the misfolding of these proteins can, when not adequately addressed, result in the onset of severe disease. Efforts to understand the structural and conformational characteristics of proteins are thus very important. Kuhar et al. demonstrated the ability of Raman spectra to distinguish between lysozyme and bovine serum albumin (BSA) based on their sequences and primary, secondary, and tertiary structural conformations, enabling the detection of small changes in protein conformation during dissolution (Kuhar et al., 2021). Nuclear magnetic resonance spectroscopy (NMR) is a powerful tool for studying protein folding that can be used to analyze both the static and dynamic structures of proteins and peptides. This technology can also elucidate detailed aspects of the structure and dynamics of large proteins. However, atomic distances and dihedral angles measured using this technique are usually time-averaged values. In addition, NMR requires a significant amount of measurement time and may require complex isotope labeling. Although methods based on molecular dynamics (MD) simulation and solid-state NMR have been developed to analyze the conformational distribution of the main-chain peptide bonds, information about the distance and angle distribution of the mean values is crucial for understanding important biological phenomena such as protein folding. Therefore, spectral methods are needed to provide additional information on protein folding and dynamics. The two classic tools of structural biology, X-ray crystallography and conventional NMR, are not suitable for studying amorphous and insoluble full-length fibers, while deep ultraviolet resonance Raman spectroscopy (DUVRR) can specifically detect protein structures in all stages of fibrosis. Protein-induced fibrosis is usually associated with human diseases such as Alzheimer’s disease. Although the conditions and epigenetic mechanisms underlying primary fibrosis are protein-specific, over 20 unrelated proteins associated with different neurodegenerative diseases are known to form fibrous aggregates. DUVRR is particularly suitable for the characterization of protein structure at the various stages of amyloid fibril formation. The combination of hydrogen deuterium exchange and DUVRR spectroscopy can detect the intersection of polymorphic fibrils with β-sheet changes in the core structure. The application of advanced statistics, chemometrics, and two-dimensional correlation spectroscopy, especially DUVRR spectroscopic analysis, significantly enhances the quality and quantity of protein structure and kinetic information. DUVRR is especially useful in the analysis of the dynamic changes occurring during amyloid fibrosis (Oladepo et al., 2012). A range of other advanced Raman imaging technologies have been designed to date, including surface-enhanced Raman spectroscopy (SERS), resonance Raman scattering (RRS), tip-enhanced Raman spectroscopy (TERS), and coherent anti-Stokes Raman scattering (CARS) strategies.

Microscopy is widely used for life sciences research focused on cellular and tissue samples. While fluorescence microscopy can reliably provide strong signals, the need for genetic modification or appropriate antibodies and fluorophores to achieve labeling can complicate analytical workflows. Confocal Raman microscopy, in contrast, is a label-free, non-destructure imaging approach that can be implemented in aqueous environments more readily than infrared microscopy. Gomes et al. found that confocal Raman microscopy and associated tools were well-suited to analyses of prokaryotic cells, eukaryotic cells, and a range of normal and cancerous tissue samples, offering valuable insight into sample characteristics (Gomes da Costa et al., 2019). Further advances in Raman spectroscopy-based technological development are expected to further revolutionize clinical work. The greatest advantage of Raman sensing technologies is their ability to derive molecular information from tissues during surgery without the need for labeling. These systems can be integrated into clinical practice quickly with good ergonomics, yielding high-quality signals even when conditions are suboptimal (DePaoli et al., 2020).

The critical point for effective and accurate diagnosis of dementia occurs in the early stages. This is especially true for Alzheimer’s disease (AD). Near-infrared (NIR) RS together with advanced multivariate statistics was used for the selective diagnosis of AD using serum samples (Ryzhikova et al., 2015). These authors analyzed data from 20 AD patients, 18 patients with other neurodegenerative dementia, and 10 healthy controls (HC). The results showed that NIR RS could distinguish patients with over 95% sensitivity and specificity. The study also demonstrated the high discriminative power of artificial neural network (ANN) classification models, with particular application to the differential diagnosis of AD, and concluded that RS, based on blood testing, may assist in clinical evaluation to effectively and accurately differentiate and diagnose AD, reduce the labor, time, and cost of diagnosis, and help screen for the development and progression of AD. In 1986, Hirschfeld and Chase described the characteristics of FT-RS by coupling a diode pumped neodymium yttrium aluminum garnet (Nd: YAG) laser with a Michelson interferometer. Due to the NIR wavelength of the laser as the excitation source, the fluorescence background of the sample was greatly reduced, thus avoiding interference with Raman spectra. By combining RS with microscopy, it is easy to analyze small blood samples, such as blood traces deposited in droplets on glass coverslips. For example, sera from healthy controls, AD patients with mild to moderate symptoms, and patients with dementia unrelated to AD were studied using confocal microscopy with RS. The serum sample was placed on a microscope slide (covered with aluminum foil) and allowed to dry, after which SERS spectra were collected under laser excitation with wavelengths of 50 mW and 785 nm. Analysis of the differences in Raman signals between the different groups using genetic variation algorithms combined with ANN showed that the diagnostic fingerprint of mild/moderate AD could be distinguished with an accuracy of 95%. The combination of CARS and microscopy could also provide the advantages of deep penetration and high spatial resolution in thick samples, making the system an effective tool for tissue imaging, especially for samples with high lipid contents, such as brain tissue (Evans and Xie, 2008). The CARS-microscopy combination could provide information on both lipids and amyloid fibrils in human AD tissue using β Raman signals and 3D images of plaque interactions without the need for any fluorescence labeling (Kiskis et al., 2015). Combining CARS with gradient refractive index lenses can be used as an alternative endoscopic technique that can overcome the limitations of superficial tissues. These techniques allow the spatial resolution of spectral information on AD brain tissue *in vivo*. For example, by combining CARS and microscopic endoscopy, nonlinear optical imaging and molecular vibration spectroscopy of neuronal membrane lipids in AD brain tissue can be obtained. The study found that the peaks of membrane lipid-related CARS signals differed between normal and AD brain tissues. In addition, the Raman intensity I2850 was about four times higher than the corresponding peak intensity in AD brain tissue (I2845), which was attributed to the high GABA content in the dentate gyrus of the diseased tissue (Lee et al., 2015).

## Applications for Raman spectroscopy in the diagnosis and study of neurodegenerative diseases

Raman spectra can be analyzed in a non-destructive manner without the need for labeling, offering insight into the internal characteristics and biochemical processes active within a given sample at the subcellular level, thus offering detailed molecular insights into complex disease-related biological processes (Xu et al., 2021). A stimulated Raman scattering microscope can be used to generate histological images of tissues of interest without labeling or destroying the sample, providing real-time histological information regarding the composition of biopsy samples within the operating room. This stimulated Raman scattering microscope setup employs two lasers to facilitate the amplification of Raman signals associated with the chemical bonds present in macromolecules including proteins, nucleic acids, and lipids that are present within these tissues (Orillac et al., 2022). The most recent progress in the targeted biomolecular imaging field has highlighted the promise of combining targeted imaging with spontaneous Raman spectroscopy or stimulated Raman spectroscopy. This flexible technology is well-suited to multi-dimensional *in vivo* imaging, providing information similar to that yielded by fluorescent imaging but without any requirement for fluorescent labeling (Azemtsop Matanfack et al., 2020). Cennamo et al. (2020) conducted non-invasive tear SERS analyses to diagnose neurodegenerative diseases including AD and other forms of dementia in an effort to design new methods to address the need for reliable and timely approaches to detecting and diagnosing these conditions.

Differences in human tear SERS spectra when comparing healthy control and mild cognitive impairment-derived patient samples were successfully detected through a multivariate analytical approach, highlighting the feasibility of detecting AD-related features. Carota et al. (2022) additionally employed Raman spectroscopy to compare samples of serum from AD patients and controls in order to design a diagnostic classification model, confirming the utility of Raman spectral analyses for the indirect diagnosis of neurodegenerative diseases based on the characteristics of biofluids. Raman spectroscopy can provide fingerprint information on all biochemical indicators in the blood. The progression of AD is accompanied by changes in serum components, leading to disease-specific changes in RS spectral features. Therefore, the changes in spectral characteristics of these biological fluids can be used for diagnostic purposes. Ryzhikova et al. subsequently explored a serum sample analysis based on surface enhanced Raman spectroscopy (SERS) to develop a fast and reliable method for the diagnosis of AD in the laboratory. This method used silver nanoparticles (AgNPs) as signal-enhancing substrates and analyzed the total biochemical components of the serum using RS. The resulting SERS spectra were analyzed using an ANN for multivariate data analysis to detect and identify AD. This method was validated by analyzing serum samples from three different groups of subjects: AD patients (n = 20), patients with other forms of neurodegenerative dementia (OD, n = 18), and healthy control group (HC, n = 10). The research results indicated that SERS analysis of the serum has great potential and can be developed further into an effective and accurate new clinical detection method for diagnosing AD (Ryzhikova et al., 2019).

Ralbovsky et al. (2019) used Raman hyperspectral analysis of saliva samples for diagnosing AD. The samples were from normal individuals, and patients with attention deficit disorder and patients with mild cognitive impairment (MCI). A concept validation study was conducted by applying genetic algorithms and ANN machine learning techniques to spectral datasets to construct diagnostic algorithms. Machine learning (sometimes referred to as multivariate statistical methods) was used to generate a classification model utilizing the differences between the spectra from the three groups and identifying patients with ADD and MCI with high accuracy. The application of machine learning to data cubes can elucidate the spatial distribution of biochemical components. As the entire biochemical composition of the sample was analyzed, the spectral characteristics of different disease states is integrated according to multiple biomarkers of the diseases following analysis of the entire biochemical composition of the sample. This significantly improves the sensitivity to the disease. In addition, due to the heterogeneity of the samples, the distribution of the biochemical components within the samples may also vary. Therefore, not all spectra collected from a single sample are typical, and each spectrum will display local concentrations of high or low biomarkers and biochemical components. Therefore, the four unknown samples detected during the external validation process were classified as belonging to the category of most spectra. The combination of RS and machine learning, through internal cross validation, could successfully distinguish between patients with overall brain damage and hypoxia and normal individuals, with an average age accuracy of 99%. External blind validation was conducted using independent datasets, further validating these results with an accuracy rate of 100%. This concept validation study demonstrates the incredible potential of Raman hyperspectrology in identifying and screening AD based on saliva analysis (Ralbovsky et al., 2019).

Many studies of dysregulated protein aggregation have been performed for over four decades given the importance of these processes in neurodegenerative disease. Aggregation-related changes in secondary structure and protein conformation ultimately shape the aggregation process and related cytotoxicity. Raman and infrared spectroscopy approaches are commonly used to assess abnormal protein aggregate secondary structural characteristics. The nanoscale spatial resolution afforded by these techniques has provided new perspectives on pathological protein aggregation (Wilkosz et al., 2020). Fueled by the design of new spectral tools and techniques, Raman spectroscopy offers a promising means of monitoring the progression of brain diseases, In some reports, Raman spectroscopy has been reported to effectively enable the classification of tissues from different regions of the brain and the detection of different brain pathology-related changes. Gualerzi et al. applied RS for the analysis of EVs as a diagnostic and predictive tool for PD. After purifying EVs from the sera of patients with PD and healthy individuals, the EVs were analyzed by RS, successfully distinguishing PD patients from the control group. A correlation between the Raman data and PD clinical scores was also demonstrated. RS analysis of EVs in the circulation can serve as a reliable, automated, and sensitive method for the stratification of patients with Parkinson’s disease and evaluating their rehabilitation and drug treatment outcomes. RS has also been used in PD research to study the structural characteristics and changes in α-synuclein, as well for analysis of patient blood samples to determine changes in biochemical parameters. Peaks related to nucleic acids, proteins, and lipids can be clearly detected in the spectra of EVs. Visual impairment frequently occurs in PD patients and research has found that RS can distinguish between normal and diseased retinas, as well as detecting molecular changes in retinal lesions associated with the disease. The accumulation of α-synuclein can also lead to neuroinflammation, metabolic disorders, and the death of photoreceptor cells. In rat sera, RS together with binary machine learning algorithms could differentiate between animals fed on standard and high-fat diets. It is known that obesity resulting from the consumption of a high-fat diet increases the risk of developing AD in the future and can trigger a pre-AD state in the brain before symptoms appear. Partial least squares discriminant analysis can identify these patients with sensitivity and specificity. These results were further confirmed through external validation and the accuracy of identifying specific groups was found to reach 100%. Genetic algorithms have shown that proteins and lipids have an impact on distinguishing between two types of serum donors. This study provides the first step in assessing the risk of individuals entering the pre-AD state. (Ralbovsky et al., 2021). A growing body of research continues to emphasize the utility and efficacy of Raman spectroscopy technology in the context of brain disease diagnosis (Ranasinghe et al., 2022). Huefner et al. first used SERS to evaluate the accumulation of mutated huntingtin aggregates in cortical homogenates and serum samples of female R6/2 mice (an HD transgenic mouse model) with specific spectral characteristics. They found significant differences in the spectral range of 400 cm^−1^ to 1,200 cm^−1^ between HD mice and the control group and demonstrated the feasibility of obtaining their spectral biomarkers through SERS in HD patients, This method may play an important role in the early diagnosis and treatment of HD (Huefner et al, 2019).

Despite the great promise that Raman spectroscopy holds as a tool for the clinical analysis of brain diseases, several barriers to clinical translation remain. Most notably, Raman spectroscopy yields a relatively weak signal, can be disrupted by the fluorescence of biological samples, and requires a prolonged collection time, extensive data processing, and high costs. Raman spectra also consist of many spectra for a complex mixture of chemicals, necessitating artificial intelligence- or other machine-based analytical strategies rather than more traditional linear approaches. While a range of dimensionality reduction methods, such as principal component analysis (PCA), have been designed, owing to the large dimensions and small sample sizes characteristic of Raman spectra, these machine-assisted analytical strategies face a high risk of model over-fitting or under-fitting. Raman spectroscopy-based approaches to rapidly and reliably analyzing clinical samples are urgently needed in order to assess cell types and to analyze the potential for drug resistance in a clinical setting (Wang et al., 2021). Raman probes are commonly used to visualize and identify biomolecules owing to their potential application for the detection of complex chemical structures, their ability to simultaneously detect several target molecules, and their value when visualizing functional molecules in cells. However, signal strength limitations continue to hamper the biological application of these probes (Watanabe et al., 2022). SERS is a valuable biomedical imaging tool, but it can also suffer from suboptimal signal-to-noise ratios resulting from overlapping Raman bands resulting from tissue complexity and the particular Raman analysis molecules used for these studies (Plakas et al., 2022).

## Raman spectroscopy in AD

**Figure 5:**
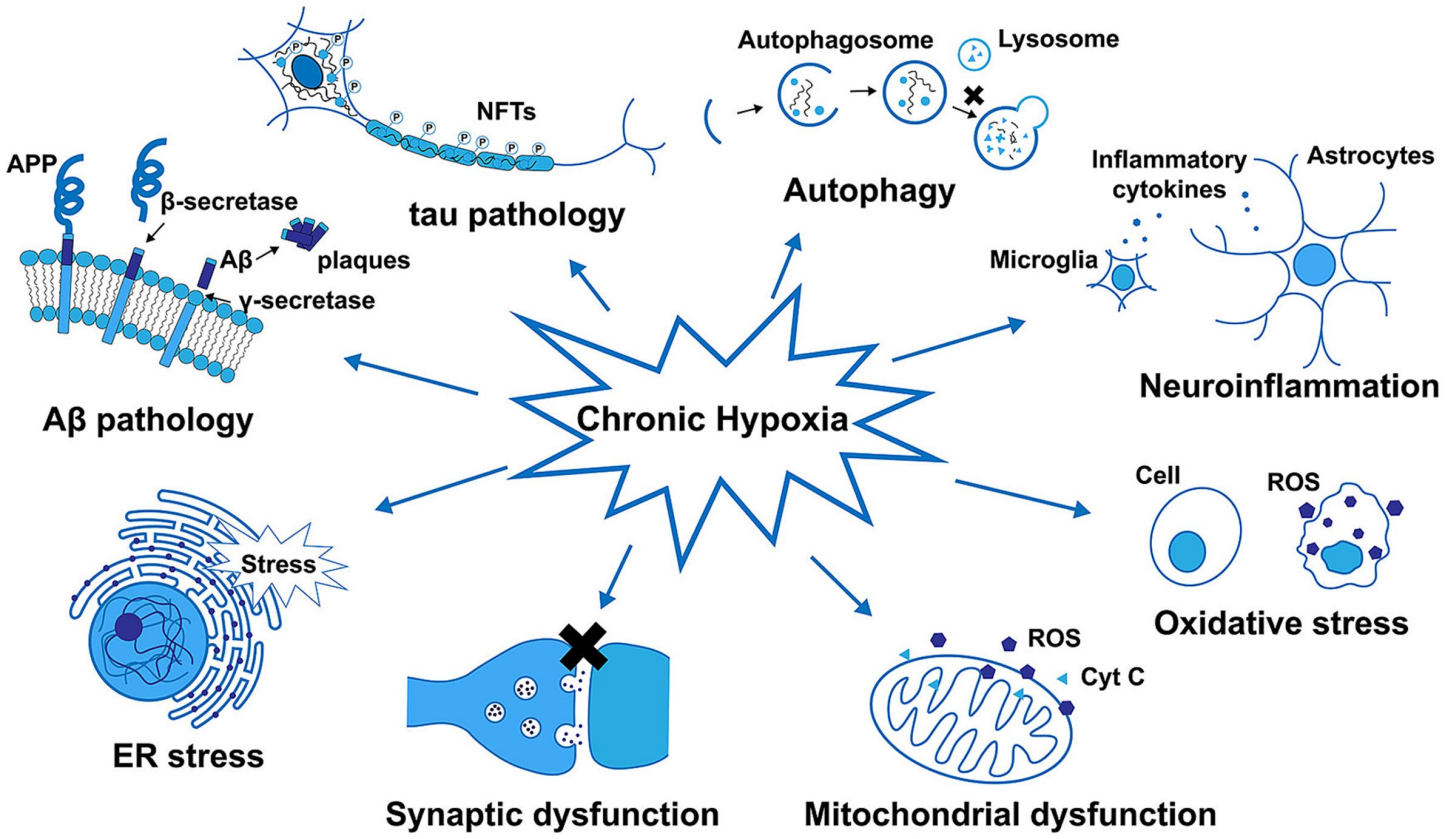
The impact of chronic hypoxia on amyloid-β (Aβ) metabolism, tau phosphorylation, autophagy, neuroinflammatory activity, oxidative stress, mitochondrial and synaptic dysfunction, and ER stress in Alzheimer’s disease (Zhang et al., 2019).

**Figure 6:**
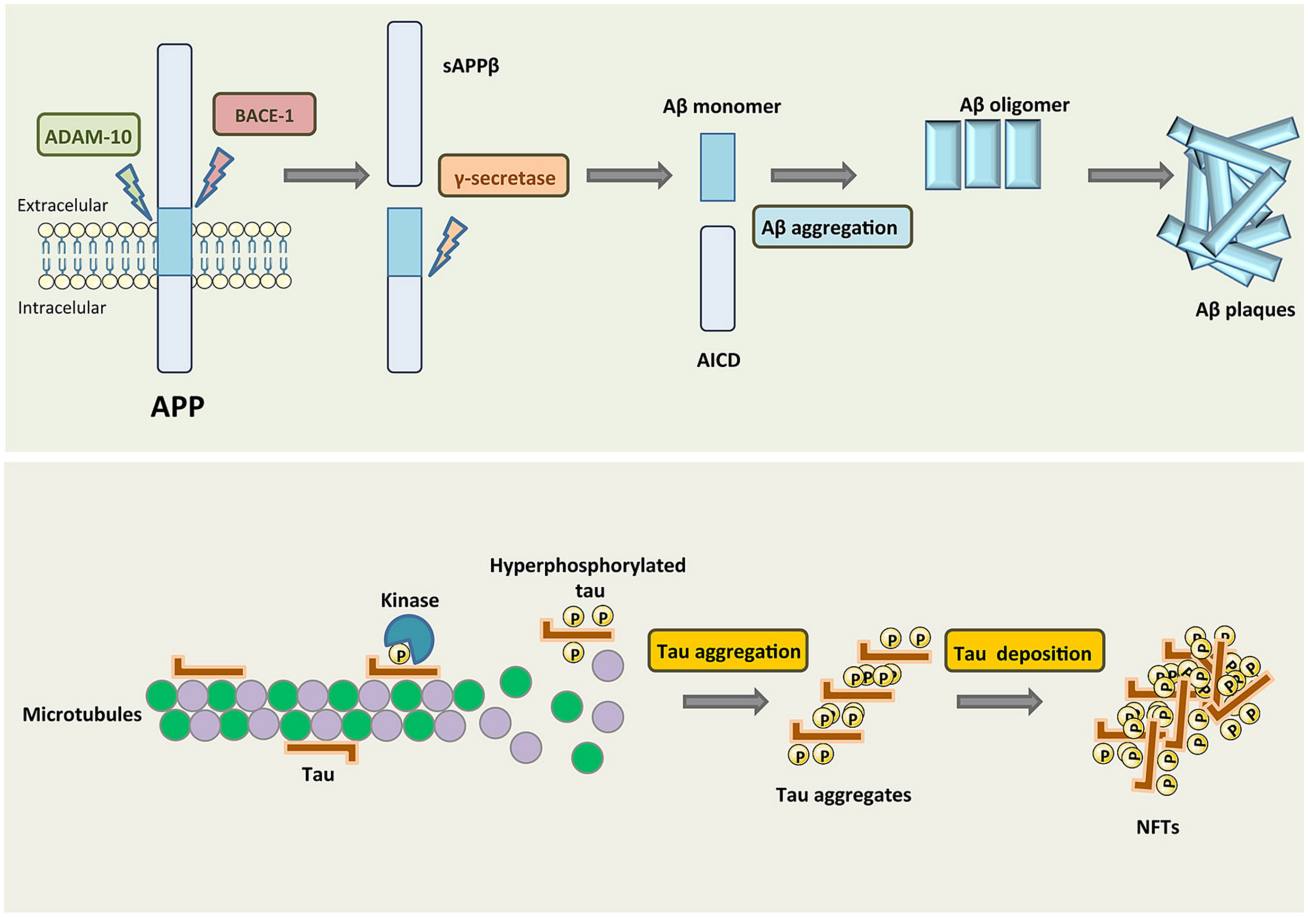
Amyloid protein pathogenesis (Baptista et al., 2014).

AD is the most common form of dementia, resulting in progressive neurodegeneration that is characterized by the accumulation of amyloid β (Aβ) protein accumulation within the neocortex and medial temporal lobe, producing neurofibrillary tangles and neuroinflammatory plaques (Breijyeh and Karaman, 2020). Exposure to chronic hypoxic conditions can impact Aβ metabolism, oxidative stress, autophagy, tau phosphorylation, neuroinflammatory activity, synaptic dysfunction, and endoplasmic reticulum (ER) stress related to the pathogenesis of AD (Figure 5). An overview of amyloid pathogenesis is provided in Figure 6, with initial shifts in the cleavage of the integral membrane protein amyloid precursor protein (APP) by β Secretory enzyme (BACE1) and γ Secreting enzyme, ultimately yielding insoluble Aβ fibrils that oligomerize, spread into synapses, and thereby disrupt synaptic signaling. The polymerization of these fibrils results in the formation of amyloid plaques and tangles, followed by microglial recruitment to the site of these plaques. The subsequent activation of these microglia provokes local inflammation and consequent neurotoxicity (Tiwari et al., 2019). Despite many phase III clinical studies having been conducted to date, efforts to diagnose AD based on the detection of amyloid plaque-related neuropathological traits remain ineffective (Lei et al., 2020).

In order to gain reliable insight into the pathogenesis of AD, the ability to detect disease-related biomarkers in the brain at early time points is essential. Accordingly, Wang et al. designed a platform for rapid AD biomarker screening through the use of graphene-assisted and machine-assisted Raman spectroscopy in the brains of transgenic AD model animals. They employed a linear support vector machine (SVM) strategy to clarify the importance of particular spectral features, thereby allowing for the more effective classification of AD and non-AD spectra based on Raman wave numbers. The resultant spectral characteristic maps enabled the determination of AD biomarkers that included Aβ, tau, and other biomolecular targets including actin, phosphatidylcholine, and triglycerides. This and other Raman machine-assisted approaches to evaluating AD may enable analyses of a range of biofluid and tissue sample types (Wang et al., 2022). Lin et al. (2022) further designed a rapid, precise, non-invasive laser tweezers Raman spectroscopy (LTRS) approach to AD diagnosis. For this study, the authors captured platelets from different 3xTg-AD model rats at different stages of disease, collecting Raman signals at a high signal-to-noise ratio via LTRS without the need for contact, enabling successful sample classification based on a combination of partial least squares discriminant analysis (PLS-DA), SVA, and PCA-canonical discriminant function (CDA) approaches. This strategy yielded respective accuracy rates of 91, 68, and 97% when distinguishing between diseases and healthy platelets at 3, 6, and 12 months of AD, indicating that this method can facilitate the diagnosis of AD throughout its progression.

The ability to diagnose AD at an early stage will enable the development of more effective preventative interventions and drugs with the potential to improve patient outcomes. The CSF is directly in contact with the brain, which is the site of initial pathogenesis in individuals who develop AD. The biochemical composition of the CSF may thus reflect AD-related pathological changes, making it the most promising biofluid for use when seeking to design a novel AD diagnostic test (Ryzhikova et al., 2021). Yu et al. (2022) developed a SERS-based approach to human CSF fingerprinting by combining the SERS strategy to facilitate the analysis of changes in the biochemical composition of CSF samples. This strategy achieved an overall 92% accuracy rate based on patient clinical diagnoses, with respective 88.9 and 100% accuracy rates among AD patients and healthy controls, respectively. A strong correlation was also detected between these fingerprints and patient clinical dementia rating (CDR) scores, suggesting that these fingerprints based on the combination of SERS and machine learning can feasibly be implemented for AD biomarker detection. Fang et al. (2019) performed a series of experiments in which they demonstrated the disruption of normal mitophagy in AD patient hippocampal samples, AD animal models, and induced pluripotent stem cell-derived human AD neurons. Higher levels of mitophagy were associated with the elimination of AD-related tau hyperphosphorylation within human neurons, reversing memory impairments in both transgenic tau nematodes and mice. The elimination of defective mitochondria is thus a key aspect of efforts to abrogate AD-related pathogenic changes. Sun et al. (2022) designed a novel SERS technique based on the combination of hairpin DNA (hpDNA) and a gold nanostar SERS probe, allowing for the quantitative detection and monitoring of levels of miR-21-5p within live cells and CSF samples *in vivo* with sensitivity levels between 1 fM and 100 pM. SERS analyses can thus enable the long-term and non-invasive monitoring of *in vivo* miR-21-5p levels, offering a powerful approach to the assessment of specific biomolecules within cells or organisms.

The neuropathology of AD is characterized by extensive neurofibrillary tangles, amyloid protein β (Aβ) deposition, and tau hyperphosphorylation within plaques. Lochocki et al. conducted a comprehensive analysis of high-resolution fluorescence images before and after staining, together with Raman mapping and stimulated Raman scattering under pre-resonance conditions, providing a means of rapidly freezing amyloid deposits within brain tissue samples from humans with AD. This enabled the fluorescent and spectral imaging and eventual thioflavin-S staining of the same sections, confirming plaque locations and correlations between the morphological characteristics of these plaques and spectral biomarkers, ultimately revealing key differences between core and fibrillary plaques. Raman maps recorded at an excitation wavelength of 532 nm revealed that the presence of carotenoids was a unique biomarker suitable for distinguishing between core amyloid plaques and non-plaque regions (Lochocki et al., 2021). Blood-based approaches to AD detection have emerged as a key diagnostic strategy, but the actual application of circulating AD-related biomarkers in clinical practice has been limited by their low concentrations and the effects of interfering proteins. Yang et al. designed a SERS-based biosensor for the quantitative measurement of tau levels in AD patient plasma samples that was capable of accurately differentiating between individuals with AD and healthy controls. This ultra-sensitive and specific SERS immunoassay platform thus offers promise as a potential tool for rapid and reliable patient evaluation in a clinical setting (Ralbovsky et al., 2021; Yang et al., 2022). Fonseca et al. employed a non-tracking spectral statistical approach to detect amyloid β in nerve tissues, with the goal of establishing the vibrational spectral signatures of Aβ aggregates as a biomarker of AD in an animal model system. Raman spectral analyses offered insight into Aβ synthesis and yielded a multi-frequency fingerprint consistent with other analytical approaches. This fingerprinting strategy for unmixed neural tissues can thus yield details images of the amyloid plaques present within the brain, providing biomarkers that can be leveraged for the noninvasive early-stage diagnosis of AD and for studies of the pathophysiology of this disease (Fonseca et al., 2019). (Carlomagno et al., 2020a,b) used SERS to analyze the sera of AD patients and healthy individuals. By introducing nanostructures to optimize the RS analysis of the serum, they induced SERS signals with more detailed and stronger Raman spectra and optimized a reliable scheme. The influence of different variables on the final spectra was considered, and multivariate analysis methods were used for analysis and comparison. The results showed statistical differences between the spectra collected from the two study groups, with accuracy, precision, and specificity of 83, 86, and 86%, respectively. Compared to other methods, the main advantage of SERS-based methods is that they are minimally invasive. By using serum, multiple analyses can be performed after diagnosis, such as monitoring the direct impact of the selected treatment or rehabilitation processes on the patient. Serum analysis can provide an overall overview of all physiological and/or pathological biochemical components, thereby reflecting pathological mechanisms. The findings of the study indicated that RS was effective for diagnosing AD and could also be used for monitoring AD progression (Carlomagno et al., 2020a,b). In AD, there is significant structural diversity in the β-amyloid aggregates, in terms of size, morphology, and localization, suggesting the phenotypic heterogeneity of the disease which may be specifically related to β-amyloid. The characterization of aggregates can classify AD into different subgroups. At present, the diagnosis of AD is based on a series of analyses involving clinical, instrumental, and laboratory results. However, changes in the signs, symptoms, and biomarkers observed in AD may overlap with those of other dementia conditions, leading to misdiagnosis. D’Andra et al. described a novel diagnostic method for AD with higher sensitivity in biomolecular detection derived from the use of a seed amplification assay (SAA), combined with the unique specificity of biomolecular recognition provided by SERS, for the innovative analysis of cerebrospinal fluid from patients clinically diagnosed with AD, mild cognitive impairment caused by AD (MCI-AD), or other neurological disorders (ONC). The results showed that data analysis using machine learning supported the SAA-SERS method to effectively identify pathological β-amyloid oligomers in the cerebrospinal fluid of AD patients suggesting that this analytical method could be used to identify specific disease features, enabling early stratification and selection of patients and improving the clinical diagnosis of AD, which is crucial for clinical treatment and pharmacological trials (D’Andrea et al., 2023).

### Raman spectroscopy in PD

PD is among the most common neurodegenerative diseases at the global level. However, most treatments for this disease are symptomatic, and no strategies are currently able to prevent the disease from progressing (Xu et al., 2019). PD is diagnosed based on symptoms of bradykinesia together with static tremors or rigidity and a medical history or physical examination without evidence of any other potential causes for these symptoms (Reich and Savitt, 2019). At the cellular level, PD is driven by dopaminergic neuron loss in the substantia nigra compacta. While the molecular etiology of this condition is not fully understood, there is strong experimental evidence from both animal model systems and human samples supporting a role for inflammation in this disease. The precise trigger that initiates the pathogenic cascade, however, remains unknown (Pajares et al., 2020). Insulin-like growth factor-1 (IGF-1) signal transduction activity is closely linked to AD pathogenesis, additionally highlighting the role that inflammation can play in the context of neurodegeneration (Wittfeld et al., 2022).

**Figure 7:**
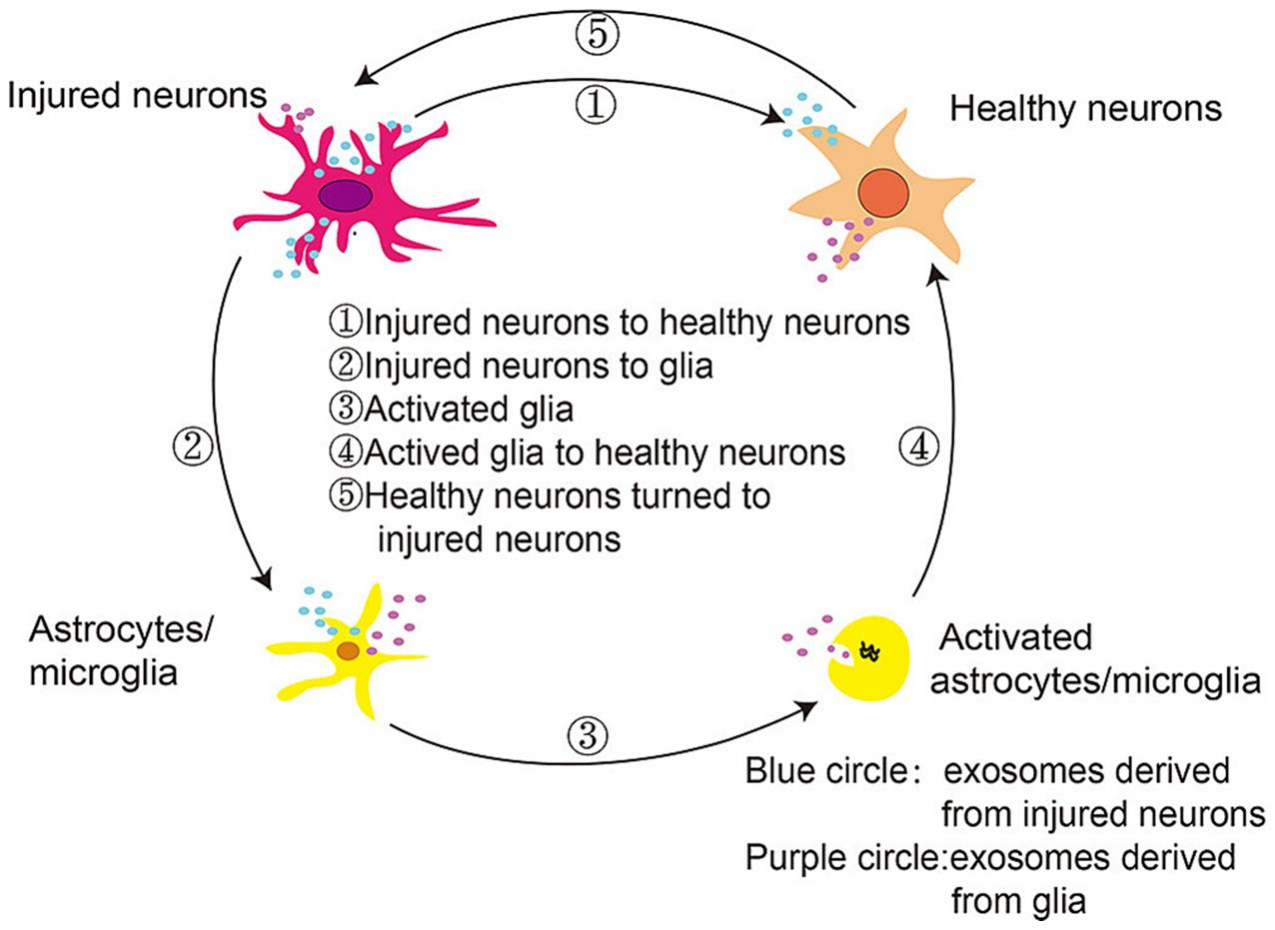
Extracellular vesicles mediate communication between neurons and glia in the context of PD progression (Yuan and Li, 2019).

As shown in Figure 7, extracellular vesicles can facilitate communication between neurons and glia during PD progression, delivering α-synuclein monomers, oligomers, and fibrils from injured neurons to healthy neurons, microglia, and astrocytes. These proteins and inflammatory mediators produced by glial cells can propagate an inflammatory response that aggravates PD-related neuronal dysfunction, spurring disease progression. While SERS can offer detailed molecular information *in vivo* that may be related to inflammation-associated changes, this technology has yet to be routinely implemented in the field of inflammatory research. SERS active nanoparticles are encoded by a unique Raman signal that is protected under various conditions and stimuli. McQueen et al. coupled gold nanoparticle clusters containing Raman-active molecules to an anti-ICAM-1 monoclonal antibody, enabling the noninvasive assessment of *in vivo* ICAM-1 expression at a sensitivity level double that of two-photon fluorescence. This advance highlights the utility of SERS as a non-invasive approach to monitoring inflammatory activity in a living system (McQueenie et al., 2012), providing opportunities to explore the link between inflammation and early PD pathogenesis.

Louis corpuscles, which primarily consist of aggregates of α-synuclein, are a hallmark of PD. Skin biopsy studies have highlighted the promise of detecting these aggregates as a diagnostic approach. Using three different techniques including *in vitro* Raman spectra from synaptophysin, biopsy samples of skin tissue from PD patients and healthy controls, and density functional theory (DFT) models, Yu et al. were able to infer the aggregation status of α-synuclein based on Raman spectra (Yu et al., 2022). This may thus represent a promising approach to PD diagnosis and treatment, consistent with the close link between the overexpression and abnormal aggregation of α-synuclein and this disease (Chen et al., 2022). Bejarano et al. used SERS to assess the presence of α-synuclein in skin biopsies from PD patients. They found significant changes in the Raman spectra of the protein. When comparing the control and PD groups, the Raman spectra changed from 1,655, 1,664, and 1,680 cm^−1^ to 1,650, 1,670, and 1,687 cm^−1^, respectively, which are related to protein aggregation. Research has shown that SERS can detect α-synuclein aggregates in the skin and thus this could be used as a minimally invasive tool for detecting the disease (León-Bejarano et al., 2022). Sevgi et al. (2021) conducted Raman spectral and microspectral analyses of the olfactory bulb from the brain of human BAC-SNCA transgenic rats and samples of colon muscular and mucosal tissue sections. Through principal and true component analyses, they confirmed the ability of Raman imaging to detect aggregates of α-synuclein, making this a viable tool for the assessment of PD-related pathological processes (Fan et al., 2020).

As α-synuclein is present at high levels in the blood, particularly in platelets and red blood cells (RBCs), Sharma et al. employed Raman spectroscopy to assess the RBCs of PD patients. These authors noted an increase in plasmin-anti-plasmin complex (PAP) levels within PD patient plasma samples consistent with fibrinolytic system activation despite the absence of any apparent differences in platelet activation following thrombin stimulation. Changes in HCy levels commonly detected in PD patients were not related to levodopa use or PAP levels, and selective gene expression analyses revealed gene subgroups related to a range of blood-related components involved in PD-related pathways, including RBCs, platelets, and fibrinolytic pathways (Sharma et al., 2021). While many PD-related biomarkers have been proposed to date, there are currently no molecules that can be leveraged to specifically detect this disease or to monitor its pathological progression in its early stages. Saliva is a complex biofluid that contains an array of biomolecules present in the blood and CSF. Optimized Raman spectrum analyses of saliva samples from PD patients can be utilized to generate classification models. Indeed, Carlomagno et al. analyzed spectral signals from salivary samples collected from 23 PD patients and healthy controls, after which they successfully developed a classification model using the Raman database. Their model achieved accuracy, specificity, and sensitivity levels exceeding 97% for individual spectral attribution, with discriminative accuracy that enabled the correct assignment of >90% of patients. The data derived from these Raman imaging studies were also closely correlated with other clinical data routinely used to diagnose and monitor PD (Carlomagno et al., 2021).

### Raman spectroscopy in HD

HD is a form of hereditary neurodegenerative disease that results in progressive neuropsychiatric symptoms, cognitive impairment, and dyskinesia. Diagnosis is generally made based on the presence of relevant clinical characteristics in patients harboring a Huntington (*HTT*) gene containing additional CAG repeats. While this condition is relatively simple to diagnose, its pathogenesis and progression can often be unpredictable, making it challenging to determine when the shift from asymptomatic carrier to disease occurs (Stoker et al., 2021). Indeed, the pathogenesis that occurs downstream of *HTT* gene mutations is complex, entailing an array of deleterious pathways that include abnormal protein fragmentation and neuroinflammation (Pan and Feigin, 2021).

**Figure 8:**
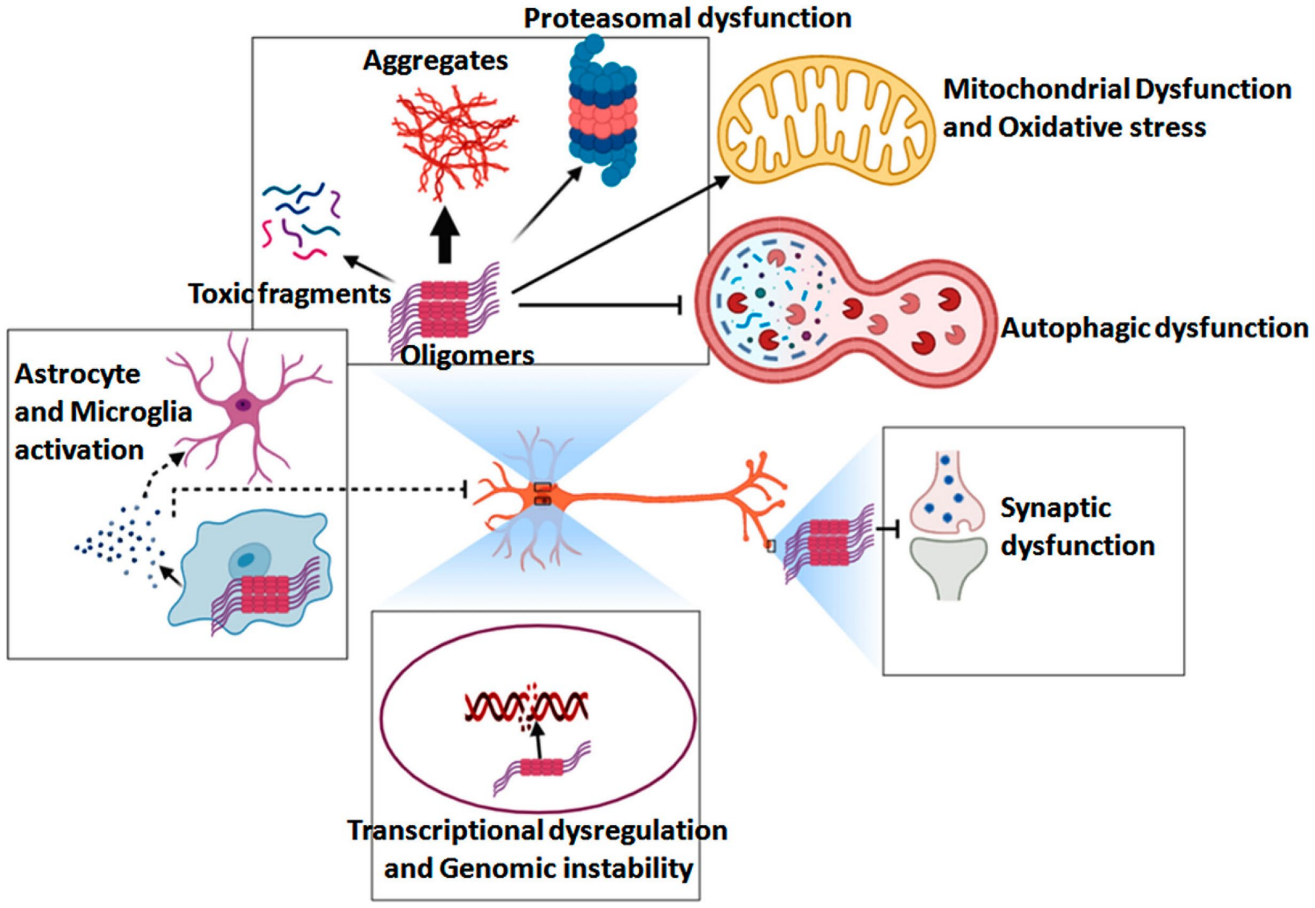
Overview of the pathogenic mechanisms that underlie HD development and progression (Ahamad and Bhat, 2022).

At the molecular level, the pathogenesis of HD is driven by toxicity from the full-length amplified Huntington protein and N-terminal Huntington fragments that are susceptible to hydrolysis-related misfolding, with abnormal *HTT* intron splicing and the somatic amplification of *HTT* gene CAG repeats further exacerbating these issues (Tabrizi et al., 2022). An overview of the pathogenesis of HD is shown in Figure 8. In general, mHTT can cause soluble HTT protein monomers to oligomerize, with the resultant aggregates interfering with normal vesicular transport and organelle function within neurons and glia. These aggregates can also disrupt a range of signaling pathways, causing mitochondrial dysfunction, proteasomal and autophagic impairment, energy deficiencies, genomic instability, and synaptic dysfunction. Many studies have focused on the development of approaches to detecting DNA mutations as a means of detecting genetic diseases at an early stage while enabling effective discrimination among clinical patient groups. SERS is widely applied in this setting as a means of detecting mutated DNA, as it can enable the detection of individual DNA molecules at a level of resolution that exceeds that of most other analytical techniques. SERS-based nanosensors are thus often regarded as promising future diagnostic tools (Pyrak et al., 2019). By using core-shell gold and silver nanoparticles as a substrate for enhancement, Zhang et al. were able to overcome limitations related to the unevenness of silver nanoparticles, thereby enabling the repeatable SERS-based detection and analysis of DNA molecules. The SERS signals corresponding to all four DNA bases were successfully identified using this platform in a non-destructive manner, and specific DNA molecule structural characteristics were also successfully characterized, underscoring the potential feasibility of applying SERS-based approaches for DNA biomolecular detection (Zhang et al., 2022).

Cytosine is often subject to epigenetic modification through DNA methylation, which can alter gene expression in a manner that can be deleterious when not appropriately regulated. UV resonance Raman analyses have been shown to provide an effective means of analyzing isolated dNTPs, dNTP mixtures, and samples of genomic DNA at excitation wavelengths of 272 nm, 260 nm, 250 nm, and 228 nm, with 228 nm serving as the wavelength best suited to the enhancement of cytosine signal. By appropriately selecting an excitation wavelength and optimizing other experimental parameters, UV Raman spectroscopy can thus be applied to detect cytosine modifications in the context of hypermethylation or hypomethylation (D'Amico et al., 2020). TERS strategies combine the chemical sensitivity of Raman spectroscopy with the nanometer spatial resolution of atomic force microscopy, offering a means of obtaining nano-scale spectral information regarding molecules of interest. Seweryn et al. (2022) sought to optimize TERS approaches for the effective analysis of DNA by preparing an atomic gold particle substrate, depositing DNA without any fixative, and optimizing the TERS probe preparation strategy. They ultimately detected a reliable DNA-TERS spectrum, offering a basis for research focused on interactions between DNA and other biomolecules involved in processes such as DNA damage repair. Huefner et al. (2019) successfully employed a systematic approach to assessing disease staging in healthy controls and HD patients based on SERS- and Raman spectroscopy-derived spectra, ultimately identifying full-range fingerprints that were both genotype- and sex-specific. SERS spectra were also found to be significantly correlated with disease progression, particularly with respect to progression from pre-positive to late HD in a manner associated with serum biomolecules, the misfolding of proteins, and nucleotide catabolism. Raman spectroscopy in general, and SERS in particular, thus holds great promise as an approach to patient grading through the tracking of HD-related spectral biomarkers. Dopamine (DA) is an important neurotransmitter in the hypothalamus and pituitary gland, and its levels are closely related to several important neurological diseases such as PD and HD. Given the important role of DA in disease regulation, developing a sensitive and reproducible DA-monitoring method is of great significance. The aim of Yu, X. et al.’s research was to develop an effective method for the quantitative monitoring of DA levels using silver nanoparticle (NP) dimers and enhanced RS. To improve accuracy and precision, a multiplication effect model of surface enhanced RS was used to analyze SERS measurements. The results indicated the potential of this detection scheme in the prevention and diagnosis of diseases associated with dysregulated DA (Yu et al., 2018).

### Applying Raman spectroscopy in other neurodegenerative diseases

**Figure 9:**
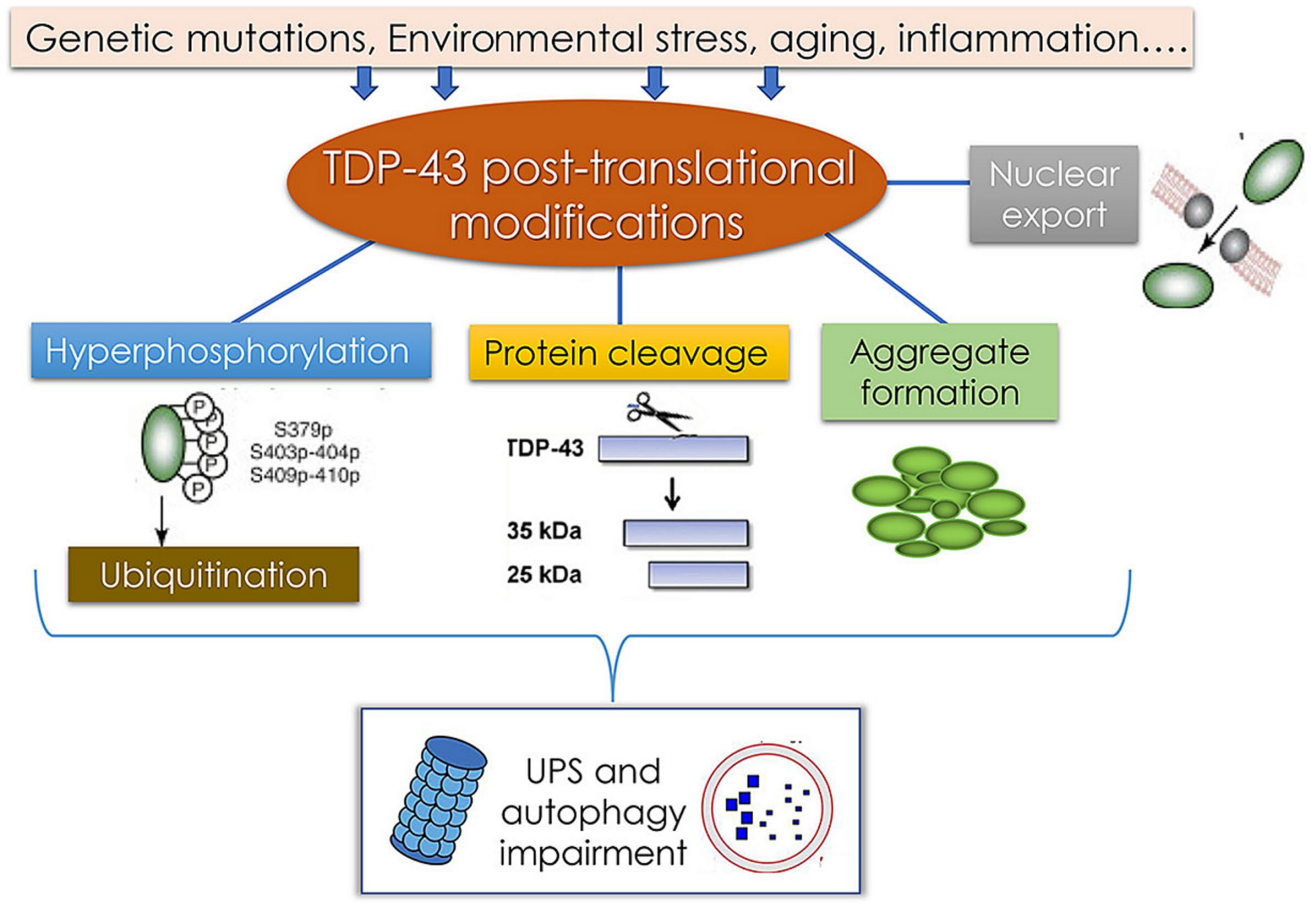
Pathological TDP-43 modification pathways and their association with the pathology of TDP-43 protein-related diseases (Palomo et al., 2019).

ALS is a progressive motor neuron disease that generally develops in adulthood and results in increasingly severe motor symptoms including muscle atrophy, weakness, and spasms. An estimated 6,000–8,000 individuals in Germany suffer from ALS, with approximately 1,200–1,600 diagnoses each year. At the molecular level, an important hallmark of ALS is the deposition of protein aggregates in the cytosol of motor neurons, with excessive TDP-43 deposition being the most commonly observed phenotype (Meyer, 2021). The pathological modification of TDP-43 and associated disease-related processes are presented in Figure 9. Factors including environmental changes, genetic mutations, and other stressors can result in abnormal posttranslational TDP-43 modification such that it becomes hyperphosphorylated, undergoes cleavage, and/or forms aggregates that are deposited in the cytosol. This, coupled with damage to the ubiquitin-proteasome system and autophagic machinery, may contribute to pathological outcomes. Despite exhaustive research efforts, the ability to reliably diagnose ALS and to gage patient prognosis remains limited. Effectively diagnosing this condition requires the assessment of phenotypic heterogeneity, the dysfunction of the central nervous system, and a range of genetic factors. As such, there is a pressing need to define novel diagnostic biomarkers that can better clarify the pathophysiological basis for this condition while also enabling the informed stratification of patients according to risk levels (Feldman et al., 2022).

Upper and lower motor neuron injury is a major driver of ALS symptoms, ultimately resulting in fatal muscle paralysis. An estimated 15% of familial cases of ALS are attributable to mutations in the *SOD1* gene, but SOD1 dysfunction is also thought to contribute to the pathogenesis of *s*poradic ALS. Mutant SOD1 misfolding and aggregation can yield toxic effects such that efforts to remediate these mutations may be an effective means of treating SOD1-associated ALS (Abati et al., 2020). Carlomagno et al. employed Raman spectroscopy as a means of analyzing saliva samples from 19 ALS patients, 10 AD patients, 10 PD patients, and 10 healthy controls, thereby optimizing parameters to achieve detailed and repeatable spectra. In multivariate analyses these authors detected significant differences among groups, indicating that this technique can be employed for disease diagnosis.

**Figure 10:**
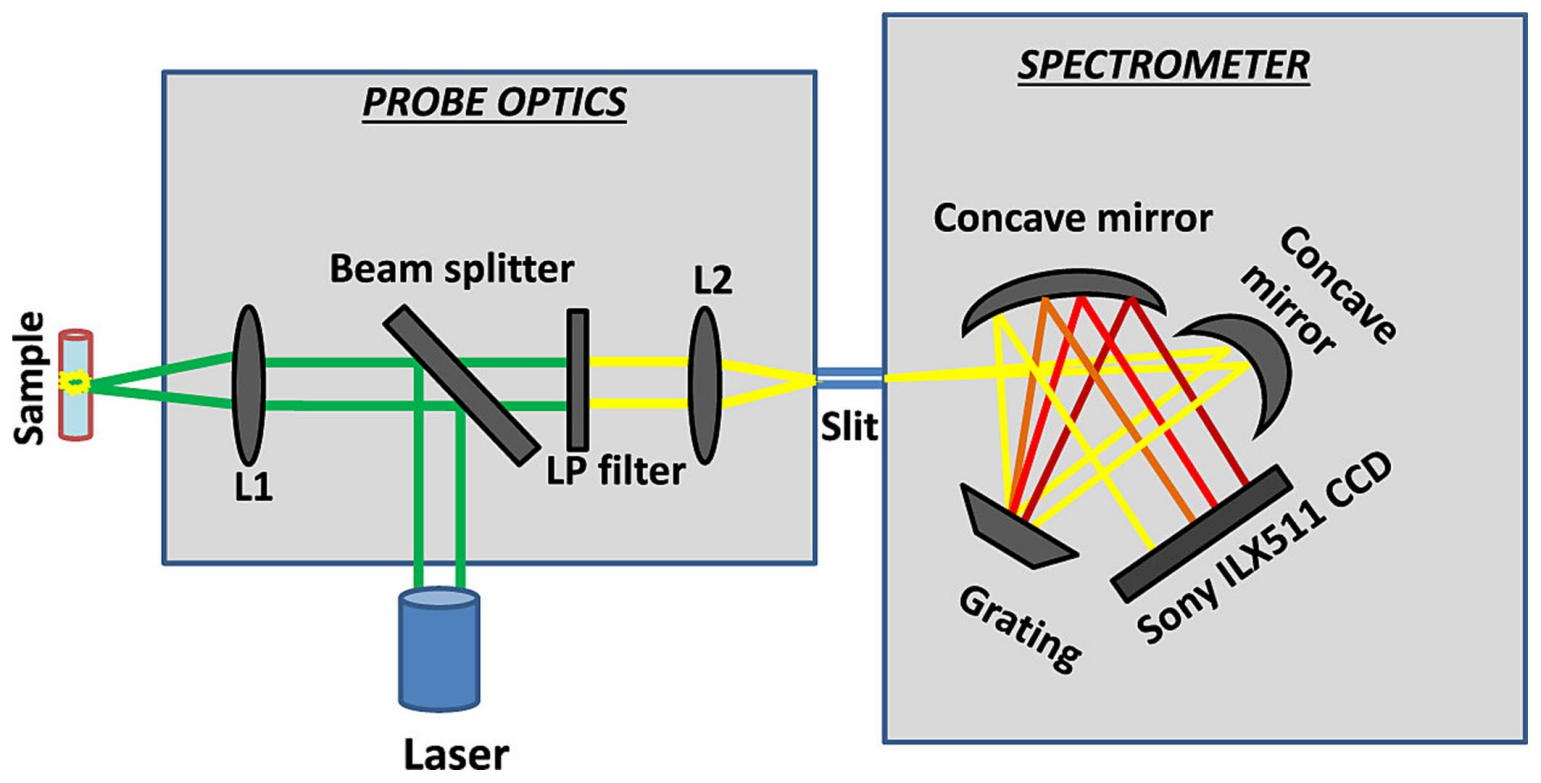
Schematic layout of the portable Raman spectrometer (Emmanuel et al., 2021).

**Figure 11:**
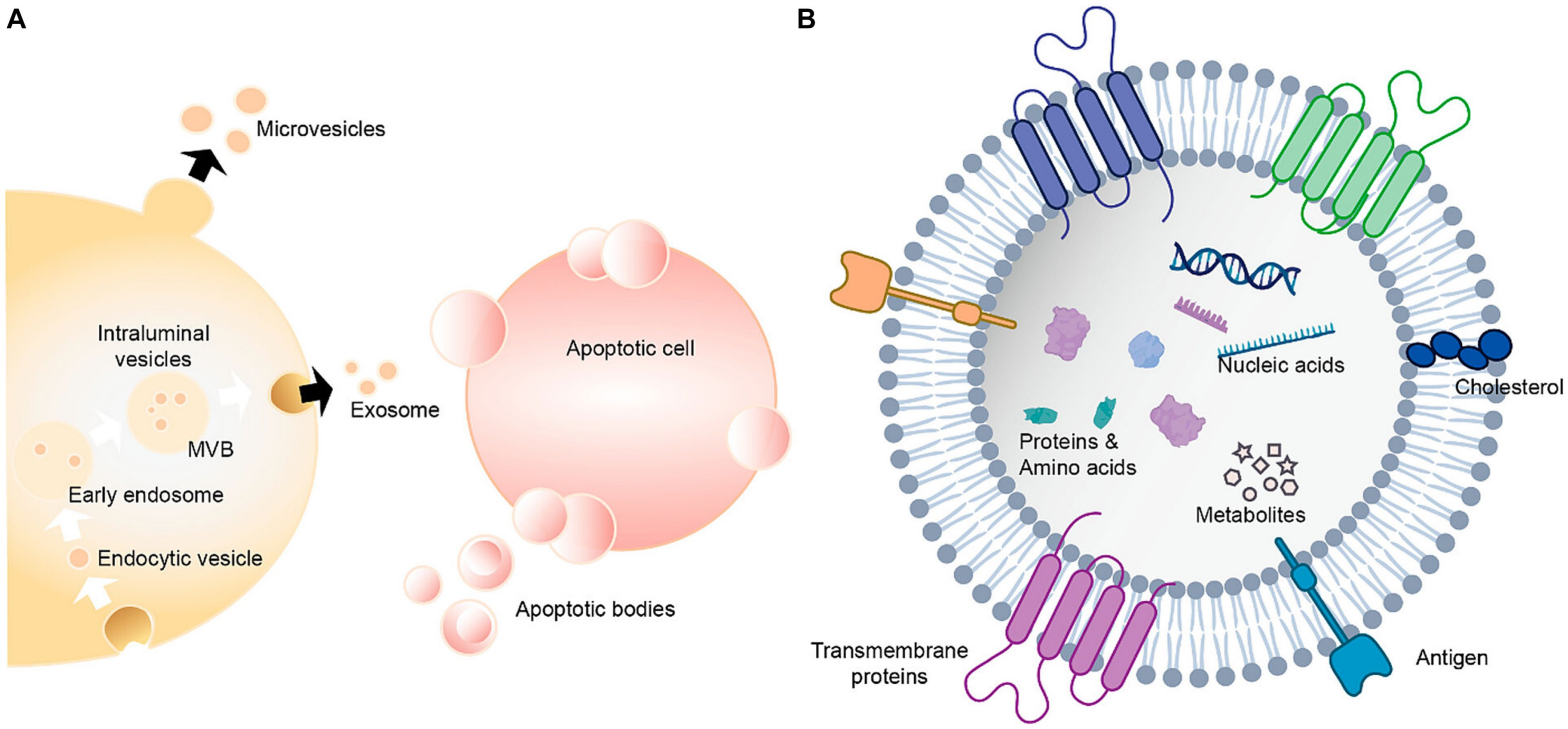
Extracellular vesicles carry a variety of proteins, lipids, amino acids, nucleic acids, and other metabolites (Qian et al., 2022). **(A)** Extracellular vesicles can be classified as exosomes, microvesicles and apoptotic bodies. **(B)** Extracellular vesicles contain nucleic acids, proteins, amino acids and metabolites.

Raman data correlations are often directly related to clinical diagnoses, highlighting key biochemical modifications that are closely associated with pathological changes. The application of this technology represents a promising means of improving the accurate diagnosis and monitoring of ALS, thereby enabling the more effective treatment and rehabilitation of affected patients (Carlomagno et al., 2020a,b). Schematic layout of the portable Raman spectrometer is presented in Figure 10. Alix et al. (2022) developed an optical fiber fluid pool to permit the spontaneous Raman spectrum-based analysis of human biofluids amenable to use outside of laboratory settings. This system was then applied to assess serum samples collected from patients with ALS on their first visit (n = 66) and after four months (n = 27). Raman spectra were analyzed through an extension of non-negative matrix decomposition known as bounded simplex structure matrix decomposition (BSSMF) that leverages raw data distributions to limit the factorization (spectral) mode. Using this system, the authors determined that the baseline Raman pattern was associated with a range of ALS-related parameters including respiratory function, symptom severity, and levels of immunity- and inflammation-related proteins including CRP and C3, with a significant spectral change between the two sample sets that was related to protein structural characteristics (*p* = 0.0002). These analyses further indicated that BSSMF implementation can reduce the necessary sample size for Rama analyses, offering pronounced advantages for the rapid quantification of disease-related alterations in samples from patients with ALS. In a separate report, Zhang et al. (2020) developed a label-free plasma SERS approach amenable use to the noninvasive detection of ALS. For their study, they recruited three ALS patient groups to examine the association between disease severity and experimental results, including patients in the ALS-1 (n = 60; ALSFRS-R ≥ 42, disease duration ≤12 months), ALS-2 (n = 61; ALSFRS-R < 42, disease duration ≤12 months), and ALS-3 (n = 61; ALSFRS-R ≥ 38, disease duration >12 months) groups. A PCA of the SERS spectrum from patient samples revealed clear differences between these three ALS patient groups and the control group. Further modeling and characteristic curves ultimately led to the identification of bands at 722 and 739 cm^−1^, and the determination that the 635–722 cm^−1^ and 635–739 cm^−1^ ratios were suitable for differentiating between ALS patients and controls. Plasma SERS analyses may thus represent a viable means of diagnosing ALS, with the bands at 722 and 739 cm^−1^ as well as the 635–722 cm^−1^ and 635–739 cm^−1^ peak ratios offering value as diagnostic biomarkers thereof. Morasso et al. (2020) analyzed Raman spectra associated with small extracellular vesicles, large extracellular vesicles, and plasma samples from ALS patients and matched healthy controls, ultimately leading to the determination that large extracellular vesicles may represent useful biomarkers that can guide ALS diagnosis. These vesicles harbor a wide array of nucleic acids, proteins, lipids, amino acids, and metabolites (Figure 11). Raman spectral analyses of these vesicles revealed ALS-related differences in lipid content compared with the aromatic amino acid phenylalanine, with a reduction in band strength, potentially representing an important finding in the ALS field.

## Conclusion and future prospects

The recent advances in the neurodegenerative disease field have highlighted the need for new technologies suitable for studying mitochondrial damage, protein aggregation, mutations, and other factors related to these diseases in an effort to enable early diagnosis and timely treatment that can prevent or delay disease onset. More advanced insight into the pathogenesis of neurodegeneration and the ability to more reliably detect it in its infancy will inform clinical efforts that may help arrest disease progression from the periphery to the central nervous system (Provenzano and Deleidi, 2021; Tansey et al., 2022).

Different types of Raman spectroscopy have proven to be accurate, effective, and flexible tools for the combined assessment of biomedical samples. These techniques are increasingly used in a range of diagnostic settings owing to their ability to specifically and sensitively detect biochemical fingerprints associated with particular cells and tissues in a non-destructive manner without the need for specific labeling (Canetta, 2021; Lin and Ye, 2021). While there are many clear advantages to Raman spectroscopy and this technology is progressively advancing toward clinical application, the full potential of Raman spectroscopy remains to be realized in the field of medical diagnostics. There are several factors underlying this fact. For one, nanoparticle use *in vivo* may be toxic, causing cellular damage or oxidative stress while also impairing the retention time of these particles and limiting their targeting potential, with their use being further complicated by a lack of clarity regarding the pathways through which they are excreted. These factors, together with the high costs, prolonged analytical times, and other challenges, continue to limit the application of Raman spectroscopy in the clinic as an alternative to more traditional diagnostics (Sloan-Dennison et al., 2021).

Despite the limitations discussed above, there are clear potential benefits to employing Raman spectroscopy as a means of diagnosing neurodegenerative diseases early in their development owing to the noninvasive nature of this technique and its ability to detect specific biomolecular fingerprints in an untargeted manner. While the Rama spectrum signal for biomolecules is relatively weak and spontaneous biological fluorescence can interfere with target frequencies, the recent advent of surface-enhanced Raman, point-enhanced Raman, confocal Raman, and laser tweezer Raman approaches provide several strategies for signal enhancement capable of overcoming these limitations in a diagnostic setting. Raman spectroscopy can allow for the highly sensitive, real-time, rapid, and non-destructive identification of trace chemicals, providing clinicians with a powerful tool for the rapid screening of pathogenic factors and the monitoring of analytes present within biofluid samples. SERS, for example, is widely utilized to diagnose HD-related gene mutations and to identify serum biomarkers of PD owing to its high sensitivity and ability to detect multiple analytes. Point-enhanced Raman spectroscopy can also enable the simultaneous assessment of changes in DNA strands such that new molecular bond formation can be detected, informing efforts to diagnose neurodegenerative diseases with unclear criteria.

In summary, this review provides a summary of the diagnostic potential of Raman spectroscopy as a means of detecting various neurodegenerative diseases. While further research will be vital to validate these findings and to translate this technique into routine clinical use, it represents an innovative, accurate, and minimally invasive strategy that is ideally suited to detecting and monitoring a range of neurodegenerative conditions from their inception and throughout the treatment and rehabilitation process.

## Author contributions

CC: Conceptualization, Writing – original draft. JQ: Writing – original draft. YL: Writing – original draft. DL: Resources, Writing – review & editing. LW: Resources, Writing – review & editing. RL: Resources, Writing – review & editing. QC: Supervision, Writing – review & editing. NS: Project administration, Supervision, Writing – review & editing.
